# Research on the Influence of Visual Factors on Emotion Regulation Interaction

**DOI:** 10.3389/fpsyg.2021.772642

**Published:** 2022-01-06

**Authors:** Zhiyong Xiong, Xinyu Weng, Yu Wei

**Affiliations:** ^1^School of Design, South China University of Technology, Guangzhou, China; ^2^Dajia Information Technology Co., Ltd., Beijing, China

**Keywords:** emotion regulation, positive emotion, emotion measurement, visual factor, extended product emotion

## Abstract

To guide the design direction of emotion regulation products that improve the positive emotions of users, investigation into the correlation between relevant visual factors and multi-dimensional complex emotions is needed. In the present study, an extended product emotion measurement method was adopted to describe the multi-dimensional emotional set of each influencing factor and calculate their weight according to the order. The positive and negative emotion indicators of all influencing factors were compared and the evaluation and ranking factors that affect users’ emotional value of emotion regulation products were analyzed. The experimental results reveal that specific emotion mapping scenes on positive emotion are the most significant among the key factors affecting user emotion. Further, the influence of emotional stickers, interactive data visualization, and text on positive emotions decreased in turn. The influence of emotional text on positive emotion was the lowest. Through investigating the visual factors that affect the psychological emotions of users, the development of emotion regulating products could be guided in a more scientific and reasonable manner.

## Introduction

According to data from the World Health Organization, over 40% of the world population do not know how to solve their emotional problems, and 50% do not take any measures to solve them. Globally, as many as one million people choose to commit suicide every year due to mental disorders. Notably, loneliness and depression in adolescence are more likely to negatively impact growth and development (Muyan et al., 2016). Thus, emotional sub-health has emerged as an urgent problem. As part of the solution for said problem, reducing negative emotions and improving positive emotions can improve work efficiency and increase the happiness of the population (Buruck et al., 2016; Pizzie and Kraemer, 2021; Roth and Laireiter, 2021).

Numerous researchers have made significant contributions to the prevention and regulation of users’ sub-health emotions. In recent years, research on affective sciences has grown considerably (Sheppes et al., 2015; Guendelman et al., 2017). Gross proposed a processing model of emotion regulation, in which emotion regulation is divided into five processes: (1) Situation selection, (2) Situation modification, (3) Attention deployment, (4) Cognitive change, and (5) Response modulation (Butler et al., 2007; Gross, 2015). Westbrook and others explored and evaluated the impact of product design on consumer sentiment (Westbrook and Oliver, 1991; Derbaix and Pham, 2004). In terms of the application of regulating products, the main focus has been on intelligent robots and humanized commodity development (Pantic et al., 2005), and the main products are physical. Currently, the daily lives of users have become increasingly dependent on the use of software products, and there has been a significant increase in the use and operating frequency of said products. However, compared with physical products, there is a scarcity of research on regulating and guiding relevant design factors in virtual products for positive user emotions (Kim and André, 2008). Since relevant elements in online products do significantly impact people’s emotions and feelings about use, the main focus of the present study was on the emotional design factors of virtual products, so as to improve product design effectiveness.

From the perspective of market research, most of the existing emotion regulation tools or products involve both doctors and patients (De France and Hollenstein, 2017). Through such products, an attempt is made to convey mental health knowledge to users faster and more conveniently or to strengthen the expression and disclosure of users’ inner feelings and demands. Such products require a high degree of professionalism, have a relatively single and fixed scene, and users must be clear about their psychological problems before use. Further, the existing products have a small range of users, and most mental sub-health populations are not covered. Most people with mental sub-health problems cannot prevent and relieve inner depression from the earlier stage. Given the aforementioned problems, the factors that affect emotion regulation in software products were mainly investigated. The product emotion (PrEmo) measurement method was optimized to help evaluate the multiple factors that can trigger positive emotions when using products.

With the developments of relevant research, researchers have begun progressively shifting attention toward research on user emotion in human-computer interaction (Jung, 2017). There are two types of emotion measurement and assessment tools: psychophysiological and self-report assessment (Nelson et al., 2017). The psychophysiological method allows for physiological responses related to emotions to be measured, such as heart rate changes or epithelioid expansion (Cuve et al., 2018). The physiological measurements can express the arousal degree of emotions in most cases, but have little impact on the activation and arousal degree of users’ emotions and will not cause strong mood fluctuations. As such, the physiological measurement method was not suitable for evaluation. In the present study, the self-report method was used to research software products and evaluate the different moderating effects of users’ emotion factors.

**FIGURE 1:**
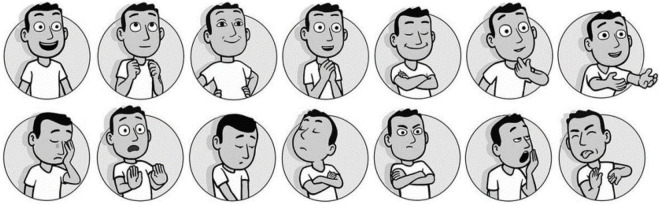
Emotional indication measurement card.

The self-report methods can be divided into the dimension method and the classification method (Betella and Verschure, 2016). In dimension-based methods, the generalized emotional state is measured rather than emotion itself. For guiding the design direction in the present study, generalized emotional states were deemed too difficult to use as a design language. Therefore, emotion was predominantly measured based on the classification method. Methods based on classification include the personal profile index (Chin, 1996), the differential emotion scale (Izard et al., 2011), and the PrEmo method proposed by Desmet et al. (2000). Through the PrEmo method, 14 kinds of emotion aroused by product design are measured, including seven kinds of positive emotion expression and seven kinds of negative emotion expression (see Figure 1). The positive emotions include desire, pleasant surprise, inspiration, amusement, admiration, satisfaction, and fascination, while the negative emotions include indignation, contempt, disgust, unpleasant surprise, dissatisfaction, disappointment, and boredom. Animated facial expressions and body movements express such emotional features. By participants selecting cartoon pictures that match their emotions, experimenters can report the emotional feelings of participants. Because emotions caused by different visual interfaces are often difficult to express in words, subjects may not have enough to express their feelings accurately, and thus, the use of non-verbal measurement was deemed better than the commonly used verbal method (Courgeon and Clavel, 2013). Subjects are asked to describe their emotional response, which requires cognitive participation and might affect the measurement. Moreover, cross-cultural language tools are also considerably complicated for accurately expressing emotions. Owing to the aforementioned factors, the PrEmo method was used to measure emotions, so as to obtain more accurate emotional data.

**FIGURE 2:**
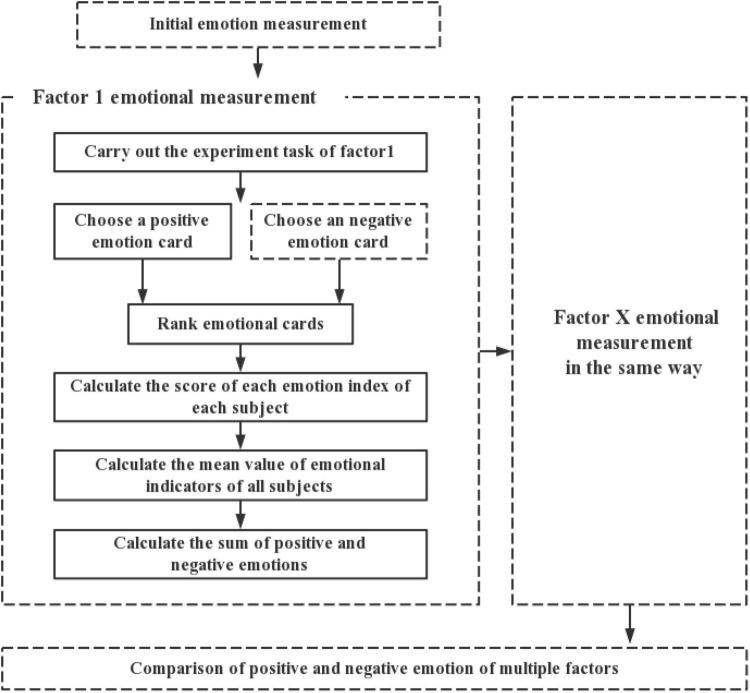
Extended product emotion (E-PrEmo) measurement process.

The classification method of emotion description traditionally involves a fixed emotion index; that is, the emotion evaluation method is based on a specific emotion. However, in people and product interaction, a product for the same user will simultaneously stimulate several emotions. At present, only one emotional indication can be considered at a time in said relationship model, which could be considered inadequate. Therefore, an expanded version of the PrEmo method was established to measure emotion, which is referred to as the extended product emotion (E-PrEmo) method. First, research on positive and negative emotions has expanded the fundamental human emotion indicators to as many as 60 kinds. As such, the screening library of emotional indicators in the E-PrEmo method was expanded. Additionally, the PrEmo measurement process was extended to the multi-angle measurement of multidimensional emotions to improve the lack of reference for a single measurement method of specific emotions. Finally, the proposed quantitative method of weight assignment (the weight is based on the ranking of emotion cards selected by the user) was used to evaluate the emotional value of different factors. The specific E-PrEmo method route is shown in Figure 2.

The E-PrEmo emotion measurement process includes: (1) refining the original emotion record of the user; (2) recording the emotional state of each factor experiment; and (3) calculating the emotional score. In the process of emotion state measurement, emotional cards (shown in Figure 1) were used to calculate the proportion of emotion values of each emotion indicator and sort the emotional states of the subjects.

In the interaction process with software products, according to many studies on the related factors affecting emotion (Beale and Creed, 2009), visual factors can occupy most of the user’s attention compared with other factors. For enhancing the emotional regulation of users in respect of visual emotion, the focus of the present study was on how the visual factors in the product will affect the emotion (positive or negative).

Online-based visual stimulation includes multiple dimensions. For example, pictures and text that convey information to website users. Several studies have revealed that emotion processors prefer graphic information rather than text information (Giese, 2001; Martin et al., 2010). Bufqin et al. also reached a similar conclusion by examining the effects of the number of photos and text length on visitors’ emotion and behavioral intention. The results revealed that more text information would lead to stress, and the enjoyment of visual processor increased significantly as the number of pictures increased (Bufquin et al., 2019). Facial emojis can express various emotions and are often used in digital and written communication. Pfeifer et al. embedded facial emojis with different emotions (happy emoticons or uneasy emoticons) in emotional fuzzy/neutral texts and asked subjects to evaluate their perceived psychological state with scores. The results showed that all facial emojis would affect the understanding of the text, and texts with happy emojis more generally conveyed positive emotions (Pfeifer et al., 2021). Generally, emojis have a more transitive meaning and can be used as a visual symbol or interpreted as non-verbal clues to realize semantic or emotional functions (Bai et al., 2019). Interactive data visualization is considerably common in wearable emotion regulation products. Users can view the data from sensors and objectively measure the psychological pressure in daily life to improve self-awareness and promote emotion regulation or behavior change. A notable experiment showed that interactive data visualization could reduce the user’s heart rate during traffic congestion (Fairclough and Dobbins, 2020). With the proposal of the meta-universe, virtual reality has attracted more attention. The three-dimensional simulation scene and immersive perception will undoubtedly produce strong visual stimulation, and many studies have confirmed the effectiveness of virtual reality technology in the field of emotion regulation (Pinheiro et al., 2021).

According to the positive emotion regulation strategy of Nélis and others, the research on the existing application products of emotion regulation, and other relevant studies, the factors that could potentially have a strong correlation with emotion were extracted, including: (a) text (Lin et al., 2012; Quan and Ren, 2016); (b) interactive data visualization; (c) emotional stickers (Nanda et al., 2012; Jaeger et al., 2017); (d) figures/geometry; and (e) virtual reality vision (Barbosa et al., 2019), and the following preliminary assumptions were proposed:

For studying the positive or negative emotion of each visual factor and the relative intensity of positive or negative emotion caused by different emotions, the following primary hypotheses were made:

Hypothesis 1: Factors a, b, c, d, e can improve the positive emotion index of users.

Hypothesis 2: The influence of each factor can be ranked as a < b < c < d < e.

## Materials and Methods

In order to test the aforementioned research hypotheses, a quasi-experimental design was adopted. The research methods are described in detail in the following sub-sections.

### Participants

Ten volunteers, five males and five females (aged 17–25, *M* = 22.4, SD = 1.5), participated in the present study. The subjects had different degrees of anxiety and had the habit of using mobile phone software for a long time. Subjects approved of all the study protocols and provided consent. The intragroup experiment method was adopted in the present study, and the experimental equipment included a recorder, an emotion card, and an interview record sheet. Each subject needed to be tested in terms of the relevant factors in the experiment. A final evaluation was conducted to comprehensively calculate all subjects’ results and make a horizontal comparison of different factors among the same experimenters.

### Procedure

Before the experiment, the current emotion of each subject was recorded, allowing for comparison after the experiment to explore whether different factors had a positive or negative effect on emotion. In the present study, 729 cases from the Institute of Positive Emotion, Delft University of Technology, were used to analyze the essential emotion perception set in the interaction between people and products (Desmet, 2012). The subjects could select, record and sort the most suitable ten emotion cards (emotion indicators). Both positive and negative cards were used to describe the current emotional state from all cards provided, as shown in Table 1.

**TABLE 1:** User emotion record before the experiment.

Order	1	2	3	4	5	6	7	8	9	10
**Emotional indicators**	Boredom	Satisfaction	Anxiety	relief	Relaxion	Indignation	Courage	Dissatisfaction	Joy	Annoyance

A relevant operable demonstration was performed to facilitate the actual operation of the subject experiments according to the visual factors. The task scenarios set by different factors are shown in Table 2. In the experiment process, with each subject as the unit, the execution order of different tasks was randomly sorted to avoid interaction between different tasks and prevent experimental errors. The experimenter provided the demonstration and allowed the subjects to conduct experience tests for several minutes. After the end of the single factor experiment, all the positive and negative emotional indicators and the reasons for the corresponding emotional indicators had to be recorded. The aforementioned experimental operations were performed on ten subjects, and each subject’s feedback was recorded in each factor experiment.

**TABLE 2:** Multi-factor experimental scene.

Factor	(a) Text	(b) interactive data visualization	(c) Emotional stickers	(d) figures/geometry	(e) virtual reality vision
**Task**	View text content that is appropriate for positive emotions	Describe the current emotional state and view the visual emotional score	View the stickers that reflects the current emotion	Describe the current emotional state and view the corresponding mood simulation graphics	View immersive virtual scene

In each factor experiment, the emotional state produced by different subjects and the intensity of other emotions would be different. To understand the proportion of emotions evoked by each factor, the emotional value had to be calculated. Assuming that in the single factor test, the total number of cards a person select to represent emotion is n, and the ranking of an emotion card in the total number is m, then (n - m + 1)/n is the weight of this emotion in the whole emotional indicators. The evaluation calculation is made with 10 as the highest score, then the score of a single emotion index is 10 (n - m + 1)/n.

## Results

The results of the present study are presented in detail in the following sections. To calculate the average value of each emotion index of a single factor in the intragroup experiment, all subjects’ emotional values were summarized. For example, as shown in Table 3, multiple emotional values corresponding to positive emotion text description factors could be obtained. The higher the emotion score, the higher the proportion of emotion in the single factor experiment, indicating that such a factor could cause more emotional responses.

**TABLE 3:** Statistical table of emotion value in singles factor experiment.

	Joy	Anticipation	relief	Courage	Relaxation	frustration	worry	Anxiety	confusion	Indignation	n
Subject 1	6.25	2.5	5	10	7.5	0	1.25	8.75	0	3.75	8
Subject 2	7	2	5	10	8	1	3	9	6	4	10
Subject 3	3	5	7	9	10	1	8	4	2	6	10
…	…	…	…	…	…	…	…	…	…	…	…
Average	5.42	3.17	5.67	9.67	8.5	0.67	4.08	7.25	2.67	4.58	

From the results of the mean calculation in Table 3, the positive emotion text could stimulate the total value Q1 of positive emotions on the subjects, where Q1 = 5.42 + 3.17 + 5.67 + 9.67 + 8.5 = 32.43. Meanwhile, the total value of negative emotions on the subjects was Q2, where Q2 = 0.67 + 4.08 + 7.25 + 2.67 + 4.58 = 19.25.

The total value of positive emotion was significantly greater than that of negative emotion. Thus, a positive emotion text could positively impact the stimulation of users’ positive emotions. According to the emotional value, all the emotions of the subjects were as follows: courage > relaxation > anxiety > trust > pleasure > uneasiness > worry > expectation > confusion > frustration.

Due to the different factors corresponding to the emotional indicators being different, in the experiment of different factors, the related emotional indicators of the experimental expression of user emotions had to be refined, statistics the top 10 emotions generated by the subjects in the process of the experiment had to be acquired, and the emotional set corresponding to different factors had to be obtained. According to each subject’s emotional value data, the corresponding mean value statistics of the emotional value of all relevant factors could be obtained. The statistical results of the mean value calculation are shown in Table 4.

**TABLE 4:** Multi-factor experimental emotion value statistics.

(a)	Joy	Surprise	Confidence	Anticipation	Relaxation	Annoyance	Dissatisfaction	Boredom	confusion	Anxiety
	5.42	3.17	5.67	9.67	8.5	0.67	4.08	7.25	2.67	4.58

**(b)**	**Joy**	**Satisfaction**	**Courage**	**Anticipation**	**Relaxion**	**Annoyance**	**Disappointment**	**Boredom**	**confusion**	**Anxiety**

	1.11	8.89	7.78	4.45	10	5.56	2.23	0	3.34	6.67

**(c)**	**Joy**	**Surprise**	**Energized**	**Fascination**	**Satisfaction**	**Boredom**	**Hate**	**Indignation**	**Distrust**	**Dissatisfaction**

	10	9	8	6	4	7	1	3	5	2

**(d)**	**Inspiration**	**Surprise**	**Courage**	**Joy**	**pity**	**Annoyance**	**Anxiety**	**Frustration**	**disappointment**	

	10	2.22	5.56	7.78	8.89	1.11	6.67	4.44	3.33	

**(e)**	**Relaxation**	**Relief**	**Dreaminess**	**Love**	**Hope**	**Joy**	**Frustration**	**Envy**	**Worry**	**Anxiety**

	10	9	8	7	6	5	2	4	3	1

**FIGURE 3:**
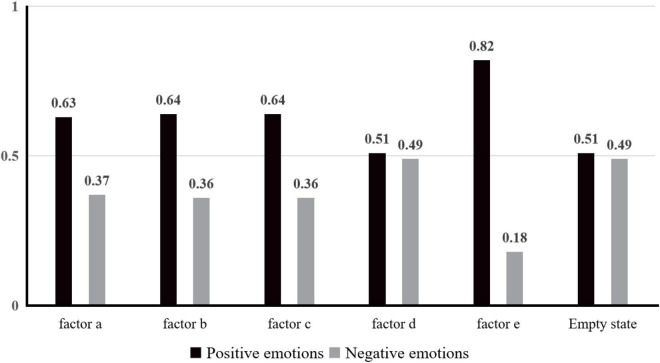
Comparison of positive and negative emotions.

The positive and negative emotion values produced by different factors were calculated according to the statistical emotion table, as shown in Figure 3.

## Discussion

Compared with the data of an empty state, findings were made that each factor positively impacted positive emotions and could weaken negative emotions to a certain extent. The results verify Hypothesis 1 that Factors “a,” “b,” “c,” “d,” and “e” can improve the user’s positive emotion indicators. As shown in Table 4, factor “a” was more closely related to anticipation and relaxation. To improve users’ expectations and desire for content, Design Factor “a” could be considered. Similarly, using an interactive data visualization to evaluate users’ emotions (Factor “b”) could increase users’ satisfaction with products and encourage users to update/use frequently. Displaying the expression corresponding to the user’s current emotion (Factor “c”) could increase the sense of pleasure and surprise and stimulate the joy of users. However, long-term negative users should be cautious and observe the negative effects of their pessimism, such as pity and stimulation. The concrete virtual scene (Factor “e”) could arouse people’s imagination through virtual simulation to instill feelings of relaxation and liberation.

According to the results in Figure 3, among all the factors studied, the effect on positive emotion could be ranked as e > c > b > a > d, which is different from Hypothesis 2. Factor “e” was the visualized virtual emotion mapping scene, which could influence the emotional feelings of users to the greatest extent and arouse more positive emotions. As the geometric figure describing emotions, Factor “d,” had a weaker influence on users’ positive emotions than other factors. There was only a slight difference compared with the situation without factors. Hence, for design that impacts users psychologically and emotionally, visual factors that positively impact the emotion of users can be achieved in virtual emotion mapping scenes, which should be adopted to maximize the design goal and save unnecessary development costs. Secondly, considering emotional stickers, interactive data visualization, and emotional text language, the design of the whole product can be improved from multiple perspectives. The present experimental research mainly collects and studies the samples of Asian young people. In the future, we will carry out a larger sample to study visual factors that affect the psychological emotions of users.

## Conclusion

Using emotion computing, the relationship between different factors and emotional indicators can be expressed by numbers. When studying a particular factor’s feedback, the causes of different emotional states can be explained more intuitively. By applying the presented E-PrEmo method to the research of emotion regulation products and expanding and quantifying, users’ emotional states in different scenarios and factors can be better recorded. Further, the emotional indicators associated with different factors can also be obtained, providing a useful reference for subsequent designs and research.

The experimental research results reveal that specific emotion mapping scenes on positive emotion were the most significant, and the influence of emotional text expression on positive emotion was the lowest. To arouse users’ emotions, the existing research achievements in emotion regulation product design can be used. Through investigating the visual factors that affect the psychological emotions of users, the development of emotion regulating products could be guided in a more scientific and reasonable manner. The present study provides further inspiration for the design process of emotional regulation products.

## Data Availability Statement

The original contributions presented in the study are included in the article/supplementary material, further inquiries can be directed to the corresponding author.

## Author Contributions

XW and YW performed the software development and organized the database. ZX, XW, and YW contributed to the conception and design of the study, performed statistical analysis and visualization, wrote the original draft of the manuscript, read, and approved the submitted version.

## Conflict of Interest

YW is employed by Dajia Information Technology Co., Ltd. The remaining authors declare that the research was conducted in the absence of any commercial or financial relationships that could be construed as a potential conflict of interest.

## Publisher’s Note

All claims expressed in this article are solely those of the authors and do not necessarily represent those of their affiliated organizations, or those of the publisher, the editors and the reviewers. Any product that may be evaluated in this article, or claim that may be made by its manufacturer, is not guaranteed or endorsed by the publisher.

## References

[B1] Bai Q. Dan Q. Mu Z. Yang M. (2019). A systematic review of emoji: current research and future perspectives. *Front. Psychol.* 10:2221 10.3389/fpsyg.2019.02221 31681068 PMC6803511

[B2] Barbosa F. Pasion R. Monteiro L. C. (2019). Attention allocation to 2D and 3D emotion-inducing scenes: a neurophysiological study. *Neurosci. Lett.* 4 165–168. 10.1016/j.neulet.2019.01.011 30630058

[B3] Beale R. Creed C. (2009). Affective interaction: how emotional agents affect users. *Int. J. Hum. Comput. Stud.* 67 755–776. 10.1016/j.ijhcs.2009.05.001

[B4] Betella A. Verschure P. F. M. J. (2016). The affective slider: a digital self-assessment scale for the measurement of human emotions. *PLoS One* 11:0148037. 10.1371/journal.pone.0148037 26849361 PMC4743948

[B5] Bufquin D. Park J. Y. Back M. Nutta M. W. W. Zhang T. (2019). Effects of hotel website photographs and length of textual descriptions on viewers’ emotions and behavioral intentions. *Int. J. Hosp. Manag.* 87:102378. 10.1016/j.ijhm.2019.102378

[B6] Buruck G. Dörfel D. Kugler J. Brom S. S. (2016). Enhancing well-being at work: the role of emotion regulation skills as personal resources. *J. Occup. Health Psychol.* 21 480–493. 10.1037/ocp0000023 26974495

[B7] Butler E. A. Lee T. L. Gross J. (2007). Emotion regulation and culture: are the social consequences of emotion suppression culture-specific? *Emotion* 7 30–48. 10.1037/1528-3542.7.1.30 17352561

[B8] Chin Y. M. (1996). Western psychological services. *J. Adolesc. Health* 19:248. 10.1016/S1054-139X(96)00178-4

[B9] Courgeon M. Clavel C. (2013). MARC: a framework. *J. Multimodal User Interfaces* 7 311–319. 10.1007/s12193-013-0124-1

[B10] Cuve H. C. Gao Y. Fuse A. (2018). Is it avoidance or hypoarousal? A systematic review of emotion recognition, eye-tracking, and psychophysiological studies in young adults with autism spectrum conditions. *Res. Autism Spectr. Disord.* 11 1–13. 10.1016/j.rasd.2018.07.002

[B11] De France K. Hollenstein T. (2017). Assessing emotion regulation repertoires: the regulation of emotion systems survey. *Pers. Individ. Differ.* 12 204–215. 10.1016/j.paid.2017.07.018

[B12] Derbaix C. Pham M. T. (2004). Affective reactions to consumption situations: a pilot investigation. *J. Econ. Psychol.* 12 325–355. 10.1016/0167-4870(91)90019-P

[B13] Desmet P. M. A. (2012). Faces of product pleasure: 25 positive emotions in human-product interactions. *Int. J. Des.* 6 1–29.

[B14] Desmet P. M. A. Hekkert P. Jacobs J. (2000). When a car makes you smile: development and application of an instrument to measure product emotions. *Adv. Consum. Res.* 13 2253–2274.

[B15] Fairclough S. H. Dobbins C. (2020). Personal informatics and negative emotions during commuter driving: effects of data visualization on cardiovascular reactivity & mood. *Int. J. Hum Comput. Stud.* 144:102499. 10.1016/j.ijhcs.2020.102499

[B16] Giese S. J. L. (2001). The influence of personality traits on the processing of visual and verbal information. *Mark. Lett.* 12 91–106. 10.1023/A:1008132422468

[B17] Gross J. (2015). Handbook of emotion regulation. *J. Am. Med. Assoc.* 298 1808–1809.

[B18] Guendelman S. Medeiros S. Rampes H. (2017). Mindfulness and emotion regulation: insights from neurobiological. *Front. Psychol.* 3:220. 10.3389/fpsyg.2017.00220 28321194 PMC5337506

[B19] Izard C. E. Woodburn E. M. Finlon K. J. Ewing E. S. K. (2011). Emotion knowledge, emotion utilization, and emotion regulation. *Emot. Rev.* 3 44–52. 10.1177/1754073910380972

[B20] Jaeger S. R. Lee S. M. Kim K. O. Chheang S. L. Jin D. Ares G. (2017). Measurement of product emotions using emoji surveys: ase studices with tasted foods and beverages. *Food Qual. Prefer.* 62 46–59. 10.1016/j.foodqual.2017.05.016

[B21] Jung M. F. (2017). “Affective grounding in human-robot interaction,” in *Conference on Human-Robot Interaction.* (New York, NY: ACM). 10.1145/2909824.3020224

[B22] Kim J. André E. (2008). Emotion recognition based on physiological changes in music listening. *IEEE* 30 2067–2083. 10.1109/TPAMI.2008.26 18988943

[B23] Lin H.-C. K. Wang C.-H. Chao C.-J. Chien M.-K. (2012). Employing textual and facial emotion recognition to design an affective tutoring system. *Turkish Online J. Educ. Technol.* 11 418–426.

[B24] Martin B. Sherrard M. J. Wentzel D (2010). The role of sensation seeking and need for cognition on Web-site evaluations: a resource-matching perspective. *Psychol. Mark.* 22 109–126. 10.1002/mar.20050

[B25] Muyan M. Chang E. C. Jilani Z. Yu T. Lin J. Hirsch J. K. (2016). Loneliness and negative affective conditions in adults: is there any room for hope in predicting anxiety and depressive symptoms. *J. Psychol.* 150 333–341. 10.1080/00223980.2015.1039474 25970325

[B26] Nanda U. Zhu X. Jansen B. H. (2012). Image and emotion: from outcomes to brain behavior. *Herd Health Environ. Res. Des. J.* 5 40–59. 10.1177/193758671200500404 23224805

[B27] Nelson B. W. Byrne M. L. Sheeber L. Allen N. B. (2017). Does context matter? A multi-method assessment of affect in adolescent depression across multiple affective interaction contexts. *Clin. Psychol.* 5 239–258. 10.1177/2167702616680061 28670504 PMC5489247

[B28] Pantic M. Sebe N. Jeffrey F. Huang T. (2005). Affective multimodal human-computer interaction. *Int. J. Hum. Comput. Stud.* 67 755–776.

[B29] Pfeifer V. A. Armstrong E. L. Lai V. T. (2021). Do all facial emojis communicate emotion? The impact of facial emojis on perceived sender emotion and text processing. *Comput. Hum. Behav.* 126:107016. 10.1016/j.chb.2021.107016

[B30] Pinheiro J. Almeida R. Marques A. (2021). Emotional self-regulation, virtual reality and neurofeedback. *Comput. Hum. Behav. Rep.* 4:100101. 10.1016/j.chbr.2021.100101 30087646

[B31] Pizzie R. G. Kraemer D. J. M. (2021). The association between emotion regulation, physiological arousal, and performance in math anxiety. *Front. Psychol.* 5:639448. 10.3389/fpsyg.2021.639448 34045991 PMC8144633

[B32] Quan C. Ren F. (2016). Visualizing emotions from chinese blogs by textual emotion analysis and recognition techniques. *Int. J. Inform. Technol. Decis. Mak.* 15 215–234. 10.1142/S0219622014500710

[B33] Roth L. H. O. Laireiter A.-R. (2021). Factor structure of the “Top Ten” positive emotions of barbara fredrickson. *Front. Psychol.* 5:641804. 10.3389/fpsyg.2021.641804 34054647 PMC8162787

[B34] Sheppes G. Suri G. Gross J. (2015). Emotion regulation and psychopathology. *Annu. Rev. Clin. Psychol.* 1 379–405. 10.1146/annurev-clinpsy-032814-112739 25581242

[B35] Westbrook R. A. Oliver R. L. (1991). The dimensionality of consumption emotion patterns and consumer satisfaction. *J. Cons. Res.* 18 84–91. 10.1086/209243

